# Genome plasticity shapes the ecology and evolution of *Phocaeicola dorei* and *Phocaeicola vulgatus*

**DOI:** 10.1038/s41598-024-59148-7

**Published:** 2024-05-02

**Authors:** Emilene Da Silva Morais, Ghjuvan Micaelu Grimaud, Alicja Warda, Catherine Stanton, Paul Ross

**Affiliations:** 1 APC Microbiome Ireland, University College Cork, Co. Cork, Ireland; 2 Food Biosciences Department, Teagasc Food Research Centre, Moorepark, Fermoy, Co. Cork, Ireland; 3 Microbiology Department, University College Cork, Co. Cork, Ireland

**Keywords:** Phocaeicola, Gut microbiome, Comparative genomics, Pangenome, Horizontal gene transfer, Microbiology, Microbiome

## Abstract

*Phocaeicola dorei* and *Phocaeicola vulgatus* are very common and abundant members of the human gut microbiome and play an important role in the infant gut microbiome. These species are closely related and often confused for one another; yet, their genome comparison, interspecific diversity, and evolutionary relationships have not been studied in detail so far. Here, we perform phylogenetic analysis and comparative genomic analyses of these two *Phocaeicola* species. We report that *P. dorei* has a larger genome yet a smaller pan-genome than *P. vulgatus*. We found that this is likely because *P. vulgatus* is more plastic than *P. dorei*, with a larger repertoire of genetic mobile elements and fewer anti-phage defense systems. We also found that *P. dorei* directly descends from a clade of *P. vulgatus*¸ and experienced genome expansion through genetic drift and horizontal gene transfer. Overall, *P. dorei* and *P. vulgatus* have very different functional and carbohydrate utilisation profiles, hinting at different ecological strategies, yet they present similar antimicrobial resistance profiles.

## Introduction

*Phocaeicola* (Bacteroides) *dorei* and *Phocaeicola* (Bacteroides) *vulgatus* are gram-negative, non-spore-forming, non-motile anaerobic rods commonly present in the human gut microbiota^1^. The genus *Bacteroides* (first described in^2^) was recently re-structured after a careful phylogenetic analysis that concluded that a number of *Bacteroides* species, including *P. vulgatus* and *P. dorei,* are phylogenetically closer to the genus *Phocaeicola* than to *Bacteroides fragilis*, the type strain of the genus *Bacteroides*^3^. *P. vulgatus* was first identified in 1933 as a common microbe in the faeces of adults, hence the name ‘*vulgatus*’ meaning common or ordinary. *P. dorei* was first isolated and characterised in 2006 from adult faeces^4^. *P. vulgatus* and *P. dorei* are indeed widely abundant and ubiquitous bacteria in the human gut^5^. They colonise the gut soon after birth in vaginally delivered infants, increasing in abundance after the introduction of solid food^1^. In caesarean section-born infants, the establishment of *Phocaeicola* species in the gut is delayed and it can take up to 18 months to match the relative abundance of *Phocaeicola* (*Bacteroides*) present in the gut of vaginally delivered infants^6–8^.

*P. dorei* and *P.vulgatus* have a wide range of carbohydrate utilisation mechanisms, as well as vitamin and hormone production genes. Different strains of *P. vulgatus* have varying effects on inflammatory diseases, including alleviating inflammation, reducing atherosclerosis, modulating the gut microbiota and regulating the levels of cytokines^9–11^. The anti-inflammatory effect of *P. vulgatus* has been associated with the production of short chain fatty acids (SCFAs) and capsular polysaccharides^9,10^. Strains of *P. dorei* have been associated with reduction of cholesterol^12^, improvement of influenza symptoms^13^, and improving atherosclerosis by reducing inflammation and lipopolysaccharide production^11^. The beneficial effects of *P. dorei* and *P. vulgatus* reported in the literature are associated with specific strains, not with the whole species, and, in some cases, the use of alternative strains could cause the opposite effect^10^. Another important consideration is that there are several studies associating *P. dorei* with metabolic and immunological conditions, such as type one diabetes^14^.

Initial 16S rRNA sequences and matrix-assisted laser desorption ionisation time-of-flight mass spectrometry (MALDI-TOF) based on β-glucosidase pointed towards the fact that *P. dorei* and *P. vulgatus* are two different species^15,16^. However, *P. dorei* and *P. vulgatus* are very similar, and there were cases of misidentification between the two species because of their mass spectra similarity^17^. Some other studies also identified strong population structure in *P. vulgatus*, with the presence of subspecies co-existing within the same subjects^18,19^. While *P. vulgatus* is closely related to *P. dorei*, a comparative genomics analysis has not yet been performed.

In the present study, we carried out a comparative analysis of *P. dorei* and *P. vulgatus* to investigate the genomic differences between these two species, their role in the human gut and their phylogenetic relationship. We included genomes obtained from whole-genome sequencing from isolates as well as metagenome-assembled genomes (MAGs) from the Unified Human Gastrointestinal Genome (UHGG)^20^ and from the Early-Life Gut Genomes (ELGG)^21^ databases. We also included genomes of isolates publicly available on NCBI^22^. We obtained a total of 3951 genomes, of which 1086 were *P. dorei* and 2865 were *P. vulgatus* genomes. We investigated genomic and functional differences, pan-genomes, phylogeny, as well as Carbohydrate-Active enZymes (CAZymes) content, antimicrobial resistance (AMR) genes, mobile genetic elements and horizontal gene transfer (HGT) events.

## Methods

### Data and metadata collection

Assemblies (MAGs from metagenomes and whole genome sequences from isolates) of the two species studied here were downloaded from the UHGG^20^ and from the ELGG^21^ databases. Genomes of isolates publicly available on NCBI (November 2022) were also included. Redundant isolates from different databases were systematically searched and removed. Metadata were retrieved from the UHGG and the ELGG databases. For NCBI isolates, metadata were retrieved using both NCBI-Datasets and Entrez v10.2^23^, searching for ‘biosample’, ‘isolation_source’, ‘host’, ‘disease’, ‘host_disease’, ‘sample_type’, ‘env_broad_scale’, ‘geo_loc_name’. We also used ffq^24^ for ENA-related metadata^25^. The metadata obtained was manually curated.

### Species identification and filtering criteria

Assemblies that were below 90% completeness and 5% contamination were filtered out using checkM2 v0.1.3^26^ in line with^27,28^. The UHGG does not differentiate between *P. dorei* and *P. vulgatus*. To assign each assembly to one of the two species, the average nucleotide identity (ANI) was calculated with the NCBI reference genomes of the two species using fastANI v1.32 and we considered a threshold higher or equal to 97.5% as the species identity threshold. Results were validated using GGDC 3.0, an in silico DNA-DNA hydridization method. All of the assemblies were assigned to a species. Additionally, the taxonomy of each assembly was checked using GTDB-tk v1.5.0^27^. Assemblies that did not correspond to their assigned species were removed. After these filtration steps, a total of 1086 *P. dorei* and 2865 *P. vulgatus* genomes were obtained.

### Genome annotation and pan-genome reconstruction

Each assembly was annotated using Prokka v1.14^29^. The annotated ‘.gff’ files obtained from Prokka were then used for the pan-genome analysis while the protein files ‘.faa’ were used for annotation with eggNOG-mapper^30^. Pan-genomes analysis were carried out using Roary v3.13^31^ with the following options: ‘-e -n -g 1000000 -v -cd 90’, in line with recommendations of^28^ for pan-genomes analysis including MAGs. Rarefaction curves for the pangenomes (total and conserved genes) were performed using the R package micropan with ‘n.perm = 100’ (100 random combinations). The pan-genomes obtained from Roary were annotated for carbohydrate enzymes using dbCAN v3.0^32^ on the CAzy database. Glycoside-hydrolase (GH) family numbers were compiled from dbCAN results, including HMM^33^ and DIAMOND^34^ using custom scripts. Later, the results were filtered to only show the GH families related to human milk oligosaccharide (HMO) genes, using both the list produced by^35^ and^36^. Heatmaps were produced using the R package ComplexHeatmap v2.10.0. Differential abundance analysis was conducted using Maaslin2 with parameters ‘transform = LOG, max_significance = 0.05, fixed_effects = species’ and default parameters otherwise^37^. For AMR genes, Resistance Gene Identifier v6.0.0 and the Comprehensive Antibiotic Resistance Database (CARD)^38^ were used on the two pan-genomes and subsequently an AMR profile for each isolate was assigned.

### Mobile genetic elements and horizontal gene transfer analysis

To look for mobile genetic elements, the ‘mobileOG-pl’ pipeline on the mobile-OG database v1.6^39^ was used, with the parameters ‘-k 15 -e 1e−20 -p 90 -q 90’. We screened for insertion sequences (IS), integrative and conjugative elements (ICEs), bacteriophages, and plasmids. For detection of putative HGTs, HGTECTOR v2.0b3^40^ was used with the option ‘-m diamond’. This tool was used on *P. dorei* and *P. vulgatus* separately, specifying their taxonomic identification number from NCBI. To identify prophages, virsorter2 v2.2.3^41^ was used, with parameters ‘-min-length 1500’. Clustered Regularly Interspaced Short Palindromic Repeats (CRISPRs) were checked using MinCED v0.4.2 with default parameters^42^.

### Phylogenetic tree

The phylogenetic tree of *P. dorei* and *P. vulgatus* was reconstructed using a multi-step approach. First, the tool PopPUNK (population partitioning using nucleotide K-mers)^43^ was used to reconstruct a draft phylogenetic tree. Briefly, PopPUNK is an annotation and alignment-free method to cluster genomes based on k-mers of variable lengths. It first estimates core and accessory genome distances between genomes by performing pairwise comparisons through k-mer matching between two sequences at multiple-k lengths to distinguish divergence in shared sequences. Then, it creates clusters using core and accessory divergences using a specified clustering model. PopPUNK v2.6.0 was used with all *P. dorei* and *P. vulgatus* filtered genomes as well as the NCBI reference genome ‘GCF_013358205.1’ for *Phocaeicola sartorii* (*i.e*., closest *Phocaeicola* species related to *P. dorei* and *P. vulgatus*) as an extra-group. The parameters ‘-fit-model dbscan’, choosing an HDBSCAN (density-based clustering based on hierarchical density estimates) model was used to find clusters in the core and accessory distances. The model was refined using ‘-fit-model refine’ and generated the draft phylogenetic tree with the function ‘poppunk_visualise’ and parameter ‘-microreact’, which creates a neighbour-joining tree from the core-distances.

GToTtree was used to obtain a coarse-grained tree to validate our approach. Briefly, eZtree finds single copy makers genes for a set of genomes and aligns them for phylogenetic reconstruction. The phylogenetic tree was obtained with Fasttree v2.1.10. The core alignment file ‘core_gene_alignment.aln’ with FastTree v2.1.10 was used to produce a phylogenetic tree (Price, Dehal, and Arkin 2010). The tree and associated metadata were plotted using R packages ‘ggtree’ v3.1.5, ‘phytools’ v0.7-90, ‘tidyverse’ v1.3.1.

### Statistical analysis

Statistical significance was calculated using Wilcoxon rank-sum test (using the function ‘wilcox.test’ as implemented in R 4.0.2, hereto referred as Wilcoxon test), with paired option. The *p* values were adjusted for false discovery using Benjamini–Hochberg procedure.

## Results

### *P. vulgatus* has smaller genomes but a bigger pan-genome than *P. dorei*

**Figure 1:**
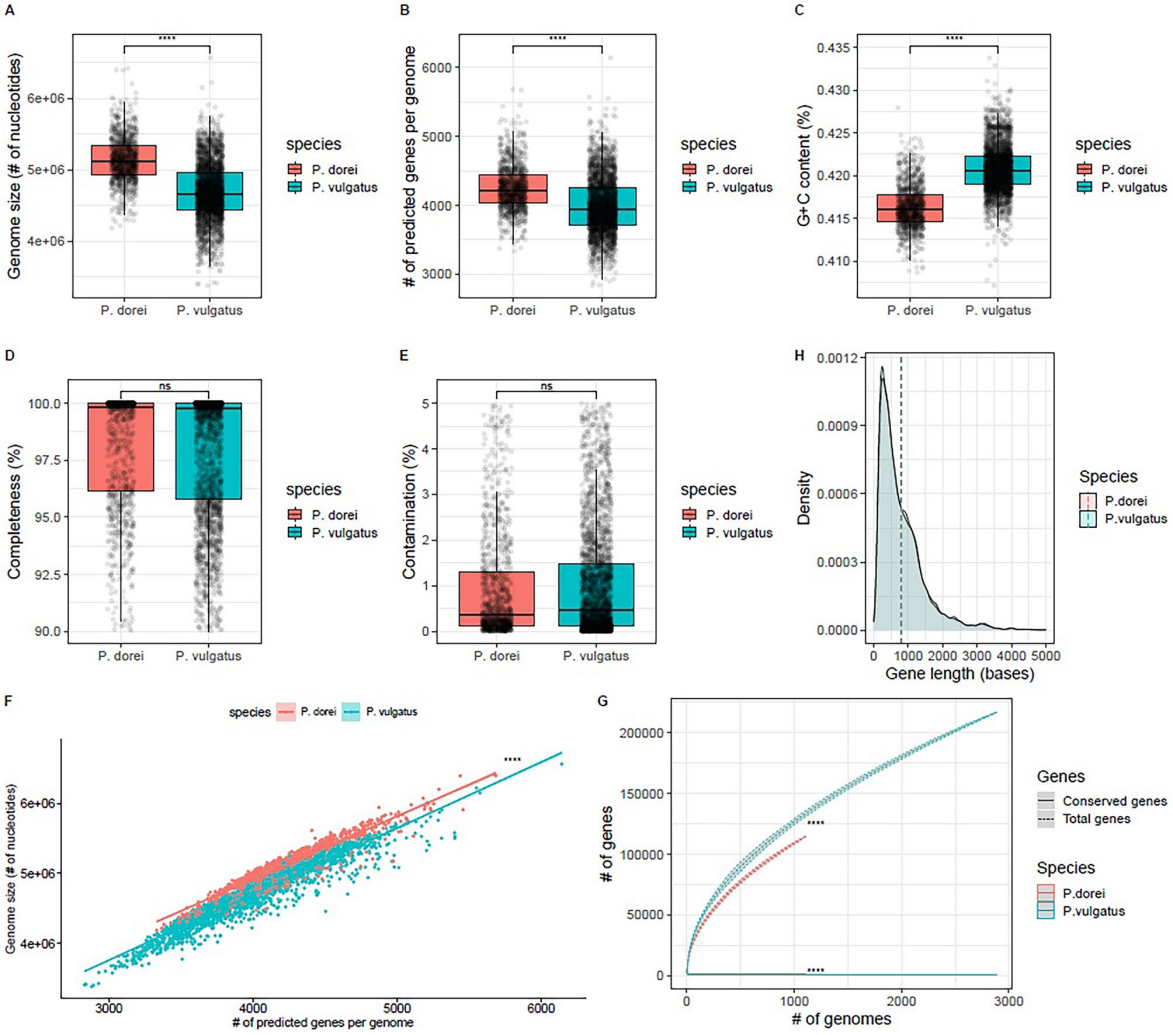
Overview of the characteristics of the genomes of *P. dorei* and *P. vulgatus*. (Wilcoxon tests, ****: adj. *p* value < 0.0001, ns: non-significant). (**A**) Genome size comparison (total number of nucleotides). (**B**) Comparison between the number of predicted genes per genome. (**C**) Comparison between the GC content (%). (**D**, **E**) Completeness and Contamination level of the assemblies used in this study (%). (**F**) Genome size as a function of the number of predicted genes per genome. The lines correspond to linear regression for each species with 95% confidence intervals (R^2^ = 0.901 and R^2^ = 0.920 for *P. dorei* and *P. vulgatus*, respectively, t-test adj. *p* values < 0.0001). (**G**) Average cumulative number of conserved genes (plain lines) and total genes (dashed lines) as a function of added genomes in the pan-genome for *P. dorei* and *P. vulgatus* (averaged over 100 random combinations). Grey area correspond to standard deviation. (**H**) Distribution of gene lengths (in bases) for *P. dorei* and *P. vulgatus.*

Assemblies and annotated genomes of both species were downloaded from the UHGG^20^, the ELGG^21^ and from NCBI^22^ (November 2022). Genomes with less than 90% completeness and more than 5% contamination were filtered out (Fig. 1D,E). ANI of more than 97.5% with respective reference genomes was used to separate *P. dorei* from *P. vulgatus*, since these species were not separated in the UHGG and ELGG databases.

The characteristics of the genomes of the two species are shown in Fig. 1A–D and Supplementary Table 1. The average genome size of *P. dorei* was 5.14 × 10^6^ nucleotides and it was significantly larger than *P. vulgatus* at 4.69 × 10^6^ nucleotides (Wilcoxon test, adj. *p* value < 0.0001) (Fig. 1A). On average, the number of predicted genes per genome was significantly higher in *P. dorei* (Wilcoxon test, adj. *p* value < 0.0001) (Fig. 1B). As expected, the number of predicted genes increased linearly with genome size for both species (R^2^ = 0.901 and R^2^ = 0.920 for *P. dorei* and *P. vulgatus*, respectively, t-test adj. *p* values < 0.0001) (Fig. 1F), but when normalizing by genome size (*i.e.*, looking at the number of genes per unit of genome length) *P. vulgatus* had more genes per unit of genome length than *P. dorei* (this corresponds to the y-intercept of the lines in Fig. 1F).

This was surprising and might indicate that *P. dorei* had more non-coding regions per genome than *P. vulgatus* and/or larger genes. Nonetheless, no statistical differences were observed in the gene-length distribution between the two species (Fig. 1H), with an average gene-length of 826.84 bases and 795.03 bases for *P. dorei* and *P. vulgatus*, respectively. The average GC content was also different between the two species, with *P. vulgatus* having a higher GC content (41.16% and 42.10% on average for *P. dorei* and *P. vulgatus* respectively Fig. 1C). Coding regions usually have a higher GC content, compatible with the hypothesis that *P. dorei* has more non-coding regions per genome.

In contrast to what was observed for the genome size, *P. vulgatus* had a larger pan-genome (*i.e*., total number of genes for the same number of genomes) than *P. dorei* (Wilcoxon test, adj. *p* value < 0.0001), with a significantly larger gene repertoire overall (114,820 genes and 217,018 genes for *P. dorei* and *P. vulgatus*, respectively) (Fig. 1G). Both pan-genomes were open according to a power-law regression (Heaps' law, B_dorei_ = 0.486, B_vulgatus_ = 0.494)^44^, reflecting the plastic nature of the two species genomes. The number of core genes was higher in *P. dorei* than in *P. vulgatus* (1968 and 951 genes, respectively), but this might be prone to artefacts as MAGs were included in this study and they are known to artificially decrease the number of core genes^28^. The larger pan-genome of *P. vulgatus* could indicate that *P. vulgatus* had more time to accumulate genes at the species level, or it could be more prone to acquiring new genes.

### *P. dorei* directly descends from *P. vulgatus* according to phylogeny

**Figure 2:**
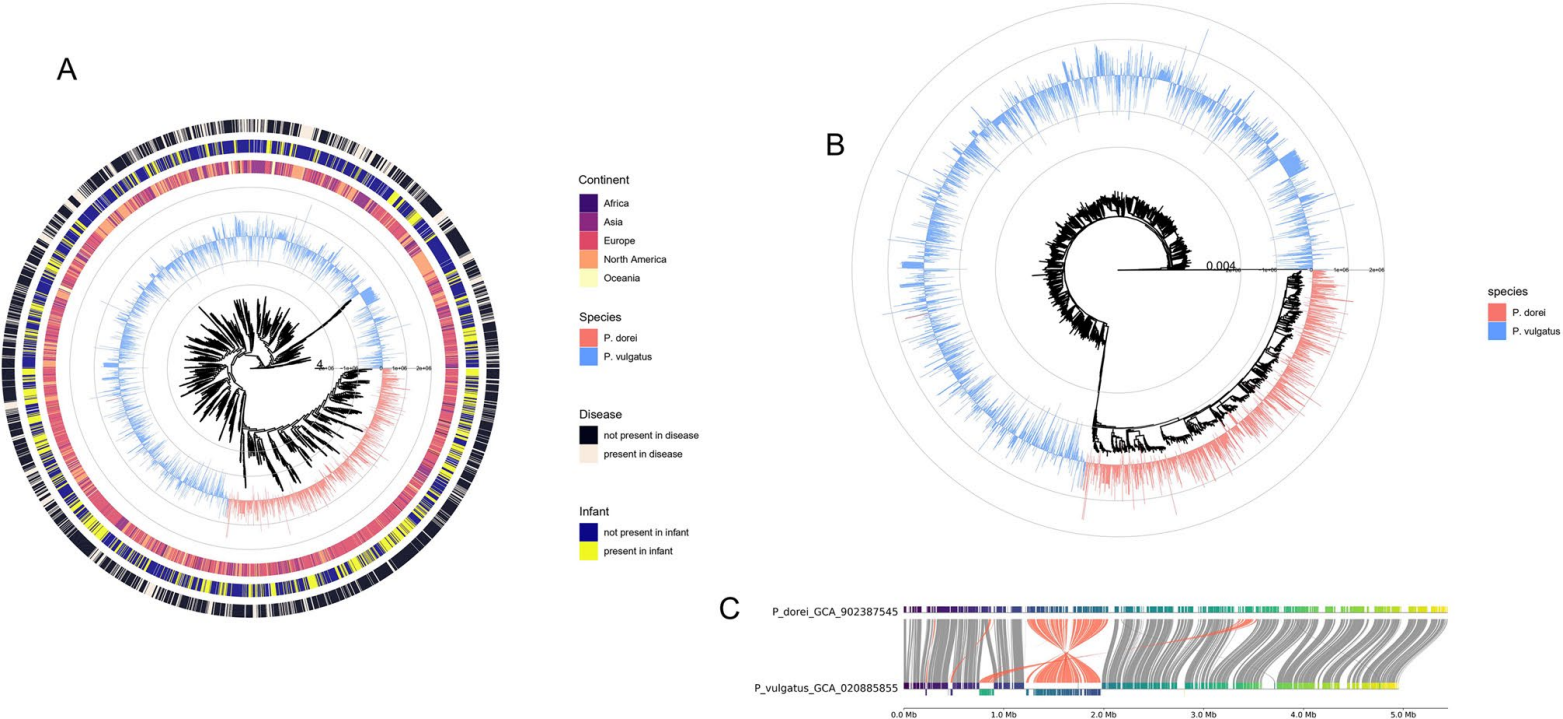
Phylogeny and genomic comparison of *P. dorei* and *P. vulgatus*. (**A**) Phylogenetic tree (natural logarithmic scale) including metadata regarding host, country of origin, isolation from disease state, isolation from infants, and including all *P. dorei* and *P. vulgatus* assemblies used in this study. The blue and red bars indicate genome size relative to the average genome size of all assemblies. (**B**) Phylogenetic tree at the normal scale displaying only the average genome size. (**C**) Comparison of synteny between *P. dorei* and *P. vulgatus* reference genomes.

To investigate the evolution and phylogeny of the two species, a phylogenetic tree including all the assemblies was generated (Fig. 2A,B). A phylogenetic tree was built using the tool PopPUNK, including the species *Phocaeicola sartorii* as an extra-group. There was a marked difference between *P. dorei* and *P. vulgatus* assemblies*. P. dorei* appeared to directly descend from a clade of *P. vulgatus.* We thus propose that *P. dorei* and *P. vulgatus* could form a unique species where *P. dorei* is a sub-species of *P. vulgatus*. This is coherent with, for example, the strong sub-species structure found for *P. vulgatus* in metagenomics data by^18^ that could be showing both *P. dorei* and *P. vulgatus*. As illustrated previously, the genome size of *P. dorei* assemblies were larger than *P. vulgatus*. Nonetheless, some clades of *P. vulgatus* appeared to have larger than average (i.e., similar to *P. dorei*) genome sizes (Fig. 2A). Whether the mechanisms for genome size increase are the same as for *P. dorei* is unclear. A subset of strains that were at the transition between *P. dorei* and *P. vulgatus* (Fig. 2B) appeared to increase in genome size across the branch of the tree leading to *P. dorei*.

Genome synteny based on the reference genomes seems to be well conserved between *P. dorei* and *P. vulgatus* (Fig. 2C). There was a large inversion/complex modification from 1.3 to 1.95 Mb, and few other minor inversions. Additional regions in *P. dorei* that were missing in *P. vulgatus* and likely responsible for genome expansion are homogeneously spread across the genomes, hinting at potential gene acquisition by HGT during evolution.

### *P. vulgatus* strains have larger genome plasticity but have fewer horizontal gene transfer events

**Figure 3:**
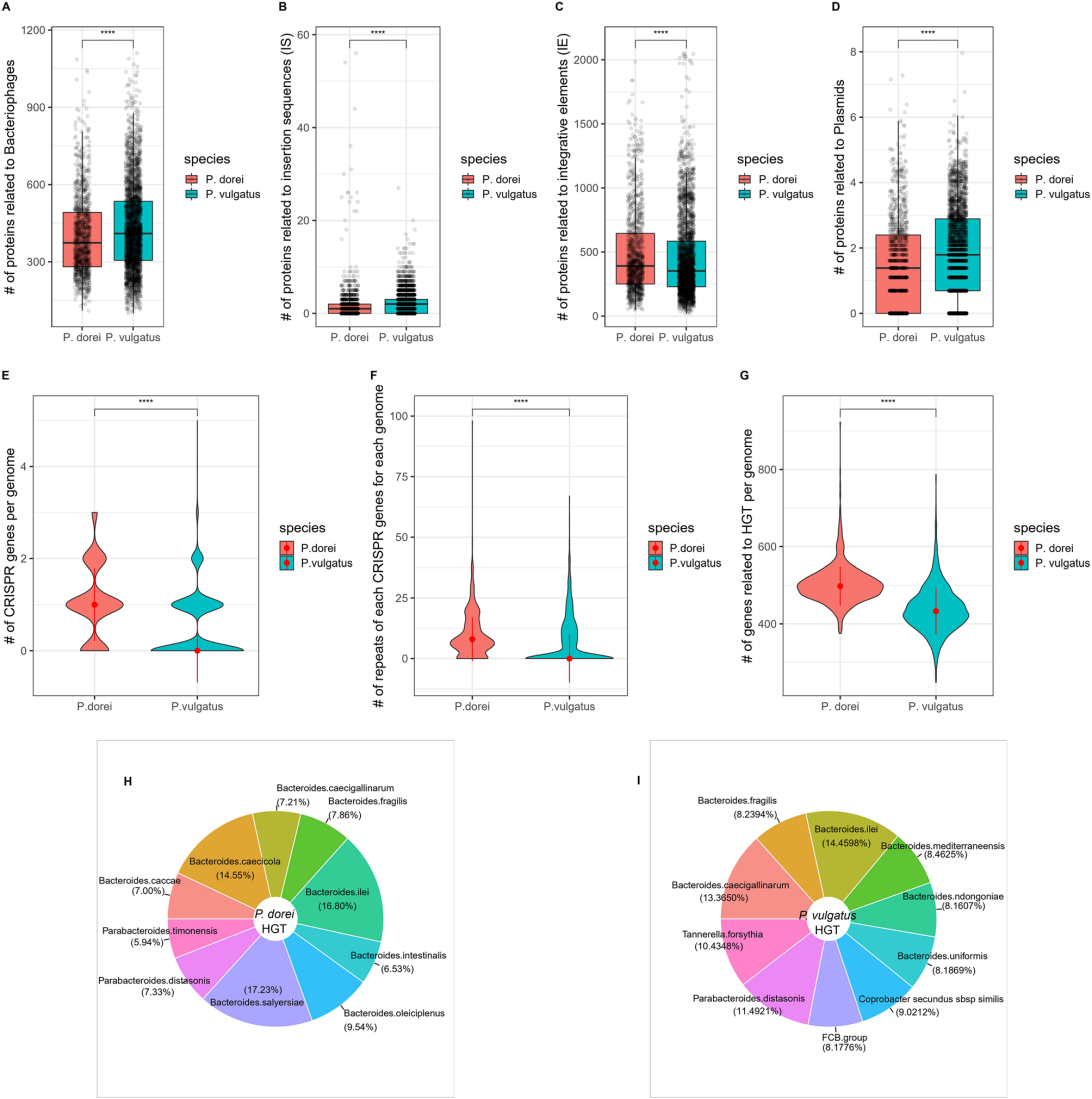
Mobile genetic elements and HGTs. *P. dorei* and *P. vulgatus* mobilome. (**A**) Number of proteins related to bacteriophage per genome. (**B**) Number of proteins related to IS per genome. (**C**) Number of proteins related to ICEs. (**D**) Number of proteins related to plasmids. (**E**) Number of CRISPR genes per genome. (**F**) Number of repeats per CRISPR gene per genome. (**G**) Number of genes associated with HGT. (H) Top 10 species associated with HGT in *P. dorei*. (**I**) Top 10 species associated with HGT in *P. vulgatus.*

To investigate the plasticity of the two species, we looked at their mobile genetic element content (*i.e.*, mobilome) and the presence of HGT events. In line with^39^, we considered different categories of mobile genetic elements: IS, ICEs, bacteriophages, and plasmids. On average, *P. dorei* had significantly less proteins associated with IS, bacteriophages and plasmids than *P. vulgatus* while it had more proteins associated with ICEs (Wilcoxon test, adj. *p* value < 0.0001, Fig. 3A–D). *P. dorei* had subsequently more genes related to HGT than *P. vulgatus* (503 and 438 genes per genome on average, respectively) (Wilcoxon test, adj. *p* value < 0.0001, Fig. 3G). For both of them, these genes were inherited from species belonging to the *Bacteroidales* order, and the majority from the *Bacteroides* genus (Fig. 3H,I). Most of the donor species were from the human gut. Of note, *P. dorei* and *P. vulgatus* had HGT coming from *Bacteroides caecicola* (5 genes per genome on average) and *Bacteroides caecigallinarum* (2.5 genes per genome on average), respectively. These species live in the caecum of the domestic chicken (*Gallus domesticus*), indicating that it could serve as a reservoir of *P. dorei* and *P. vulgatus* species where HGT can occur.

To take a closer look at potential anti-phage defense systems, CRISPRs were identified using MinCED. *P. dorei* had significantly more CRISPR systems per genome than *P. vulgatus*, with averages of 1 and 0.47, respectively (Wilcoxon test, adj. *p* value < 0.0001) (Fig. 3E). *P. dorei* also had significantly more repeats per CRISPR system per genome than *P. vulgatus*, indicating a larger use of this anti-phage defense system (9.77 and 6.96 repeats, respectively; Wilcoxon test, adj. *p* value < 0.0001) (Fig. 3F). This could also be indicative of a stronger innate immune response (*e.g*., prophage integrated signals) mediated by CRISPR systems.

### *P. dorei* and *P. vulgatus* are functionally different

**Figure 4:**
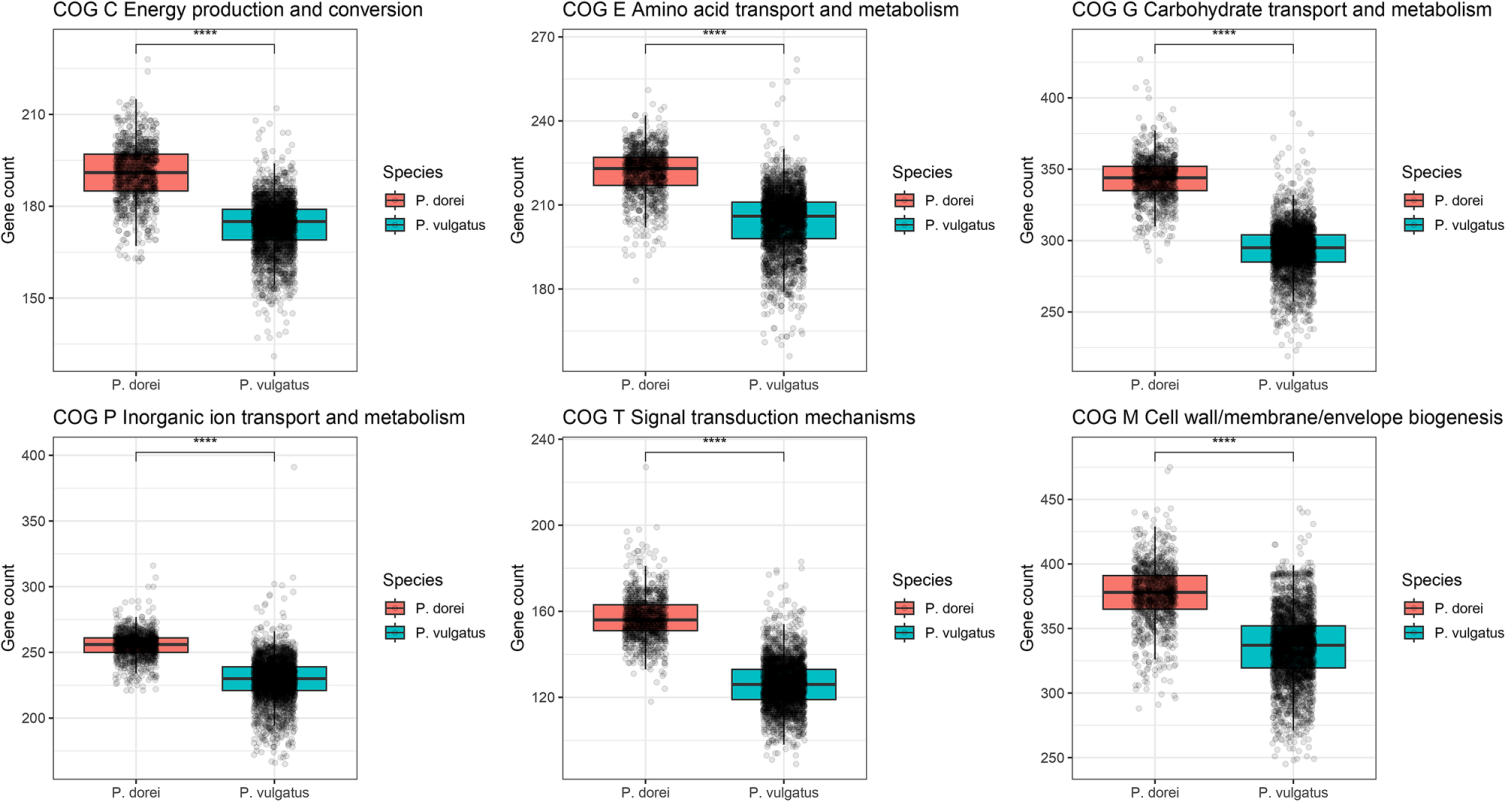
COG categories comparison for *P. dorei* and *P. vulgatus* (****, Wilcoxon test, adj. *p* value < 0.0001. (***, Wilcoxon test, adj. *p* value < 0.001).

To gain more insights into the functional differences between *P. dorei* and *P. vulgatus*, we annotated the genomes using eggNOG-mapper and extracted the Clusters of Orthologous Groups (COGs) categories for each gene. We then compared *P. dorei* and *P. vulgatus* for each category. Out of the 25 COG categories identified, 19 were differentially more present in *P. dorei* than *P. vulgatus* (Wilcoxon test, adj. *p* value < 0.0001) (Fig. 4). This difference remained true when normalising by the genome size. In particular, COG categories C (energy production and conversion), E (amino-acids transport and metabolism), G (carbohydrate transport and metabolism), P (inorganic ion transport and metabolism), T (signal transduction mechanisms) and M (cell wall/membrane/envelope biogenesis) were more present in *P. dorei* than in *P. vulgatus*. However, all these differences are related to central metabolism and could be caused by genome expansion.

### *P. dorei* and *P. vulgatus* strains have distinct carbohydrate enzyme profiles, hinting at different yet overlapping ecological niches and strategies

There is a high diversity of carbohydrate molecules in the human gut. Bacteria use carbohydrates as a carbon source, for attachment to the host, or other functions during infection (e.g., immunomodulation). CAZymes such as GH, glycosyl transferases, polysaccharide lyases and carbohydrate esterases, are enzymes involved in the assembly, modification and breakdown of carbohydrates^45^. The amount and variety of CAZymes present in a given organism can be used as an indicator of adaptation to and fitness in a certain environment. Carbohydrate utilisation is an important factor driving bacterial evolution, as it is associated with the niche each organism occupies and how well it can adapt to environmental changes. To investigate the CAZymes present in *P. dorei* and *P. vulgatus*, all the genomes collected using dbCAN v3.0^32^ were annotated on the CAZy database.

**Figure 5:**
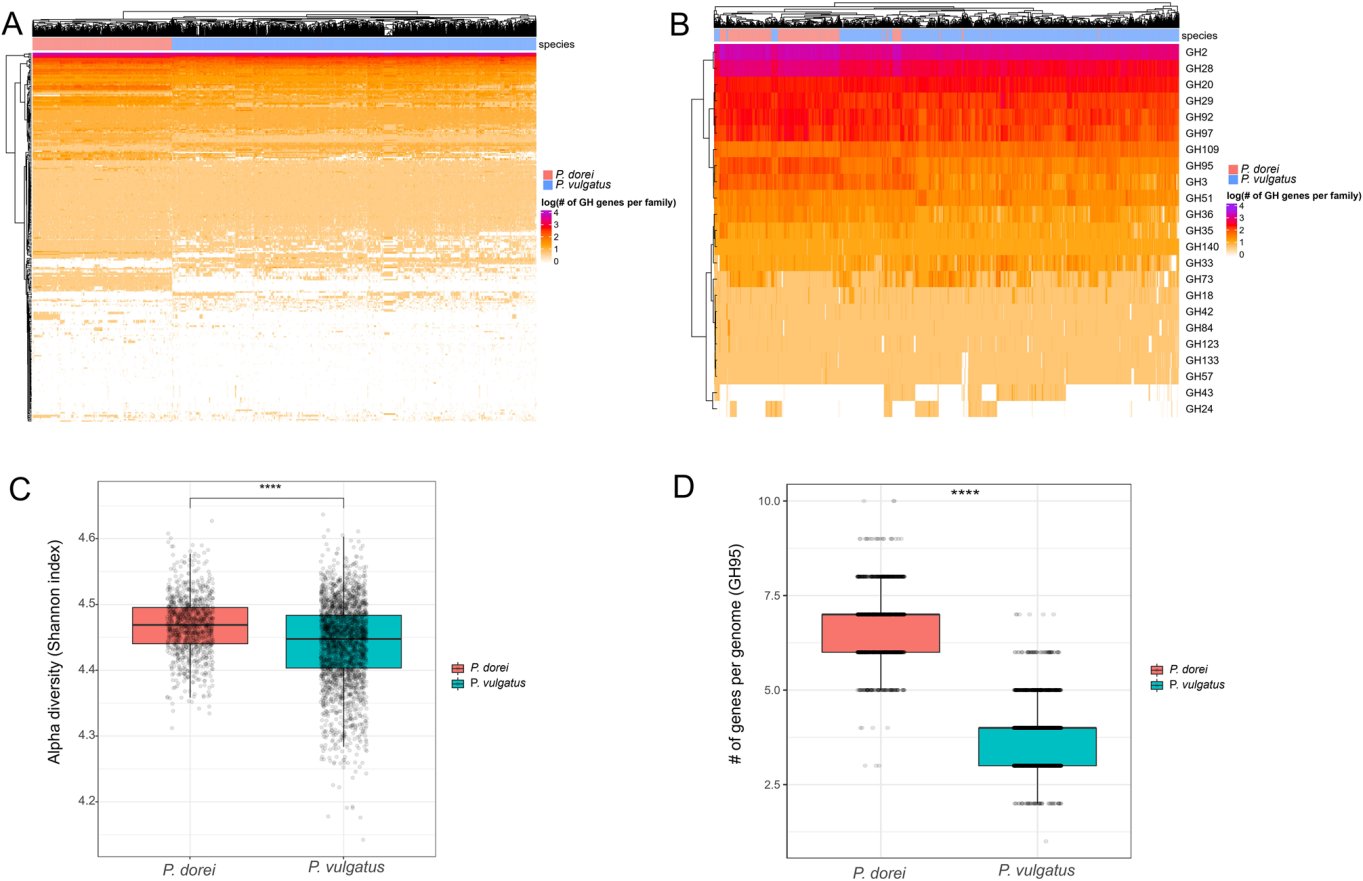
Carbohydrate enzyme profile of *P. dorei* and *P. vulgatus*. (**A**) Heat map showing the different CAZymes present in each assembly, with *P. dorei* and *P. vulgatus* genomes grouping separately (only GH families present in more than 4 genomes are shown here). (**B**) Heat map showing only GH families associated with HMO utilization genes according to^36,46^. (**C**) Alpha diversity (Shannon index) of *P. dorei* and *P. vulgatus* GH families profile. (**D**) Number of genes per genome belonging to the GH family GH95 (1,2-α-L-fucosidase) (****, Wilcoxon test, adj. *p* value < 0.0001).

When looking at the CAZymes profile of each assembly, a clustered heatmap (hierarchical Ward-linkage clustering based on the Pearson correlation coefficients) showed that they all cluster according to the species they belong to, with only 2 exceptions, thus indicating a clear separation in terms of GH profile between *P. dorei* and *P. vulgatus* (Fig. 5A). In particular, the GH families GH144 (specific to β-1,2-glucan), GH88 (unsaturated glucuronyl hydrolases), GH146 (β-arabinofuranosidase) were more associated with *P. dorei* while the GH families GH101 (specific to glycoproteins, especially mucins), GH130 (acting on β-mannosides), GH110 (active on blood group B oligosaccharide) were more associated with *P. vulgatus* (differential abundance analysis using Maaslin2, adj. *p* value < 0.05). This marked CAZyme signature difference in *P. dorei* and *P. vulgatus* suggests that both species occupy a different yet overlapping ecological niche for carbohydrate utilisation and/or ecological strategies. *P. dorei* had a higher alpha-diversity (Shannon index) of GH families per genome than *P. vulgatus* (Fig. 5B, Wilcoxon test, adj. *p* value < 0.0001), possibly indicating a higher degree of adaptability to complex carbohydrates-rich environments. Additionnally, strains isolated from or present in disease states presented a lower alpha-diversity of GH families compared to strains not isolated in disease states in *P. vulgatus*, but not in *P. dorei* (Supplementary Fig. 1, Wilcoxon test, adj. *p* value < 0.05).

*P. dorei* and *P. vulgatus* are among the species in the *Phocaeicola* genus that colonise the human gut soon after birth in vaginally delivered infants^1^. The ability to digest HMOs is an important advantage to colonise the gut of breastfed infants since HMOs are the third most abundant component in breast milk and they cannot be digested by the host^36,46^. Recently, specific GH families associated with HMO utilisation were identified in *P. dorei* using transcriptomics^36^. Using this list of GH families, we investigated the most abundant GH families related to HMO utilisation present in both species (Fig. 5C). *P. dorei* had a higher number of these GH families associated with HMO utilisation. The GH families GH2 (beta-galactosidase), GH28 (polygalacturonases), GH29 (1,3/1,4-alpha-fucosidase), GH92 (exo-acting α-mannosidases), GH97 (α-glucosidase and α-galactosidase), GH95 (1,2-alpha-L-fucosidase), GH3 (exo-acting β-D-glucosidases and α-L-arabinofuranosidases), GH51 (L-arabinfuranosidases), GH36 (α-galactosidase and α-N-acetylgalactosaminidase), and GH35 (β-galactosidases) were more abundant per genome in *P. dorei* (Wilcoxon test, adj. *p* value < 0.05). Conversely, the GH families GH20 (lacto-N-biosidase), GH109 (α-N-acetylgalactosaminidase), GH33 (sialidase) and GH43 (α-L-arabinofuranosidases) were more abundant per genome in *P. vulgatus* (Wilcoxon test, adj. *p* value < 0.05). It seems that *P. dorei* is more adapted to HMO utilisation in the infant gut microbiome, with for example more genes associated with alpha-fucosidase enzymes which are widely used by bifidobacteria to feed on the widely available 2′-fucosyllactose^47^ (Fig. 5D). However, the HMO utilisation mechanisms in *Bacteroides* and *Phocaeicola* are different than the well-characterized mechanisms in *Bifidobacterium* and not necessarily associated with specific GH families, therefore more studies are needed to fully understand *Bacteroides* HMO utilisation genes. For example, it has recently been shown that GH33 is used by *P. dorei* for utilisation of sialylated HMOs^48^. As there are more GH33 genes in *P. vulgatus*, *P. dorei* and *P. vulgatus* could have different HMO utilisation strategies. The difference could also be due to genome expansion in *P. dorei*.

### *P. dorei* and *P. vulgatus* strains have close but different antimicrobial resistance genes (AMR) profiles

**Figure 6:**
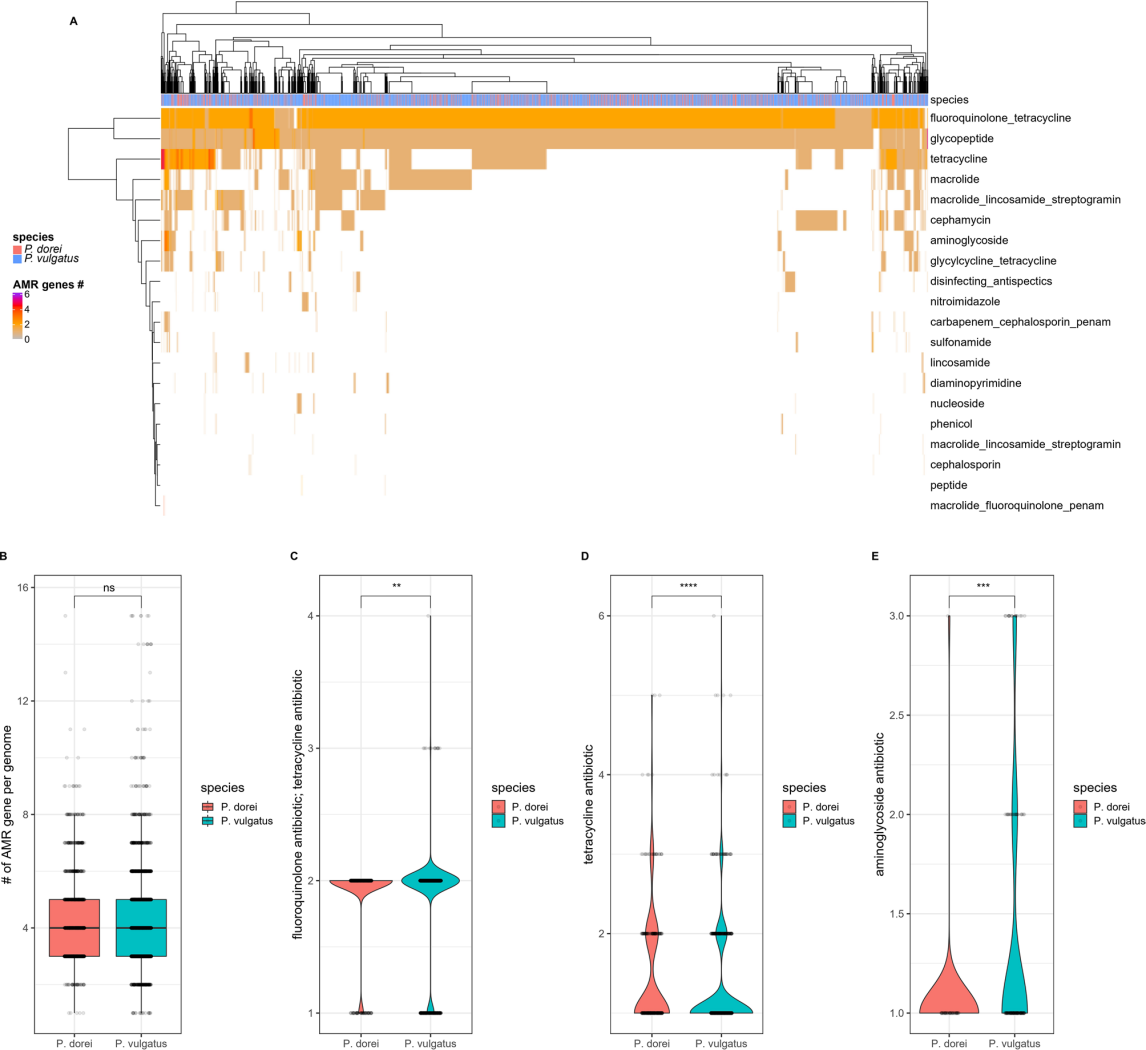
Antibiotic resistance profile of *P. dorei* and *P. vulgatus*. (**A**) Heat map of *P. dorei* and *P. vulgatus* assemblies showing the different antibiotic resistance genes present in each strain. (**B**) Comparison of the number of AMR genes present in the genomes of *P. dorei* and *P. vulgatus*. (**C**–**E**) AMR families with different abundances per genome in both species (Wilcoxon test, ****, adj. *p* value < 0.0001, ***, adj. *p* value < 0.001, **, adj. *p* value < 0.01, *ns* non-significant).

To assess the AMR genes of both species, Resistance Gene Identifier (RGI) and the CARD database were used. A total of 23 AMR families were identified (Fig. 6A), with a round average of 4 AMR genes per genome in both species (Fig. 6B). The most abundant resistance genes were fluoroquinolone and/or tetracycline, glycopeptide, macrolide. *P. vulgatus* and *P. dorei* assemblies do not cluster separately according to AMR genes present on their genomes when looking at a clustered heatmap (hierarchical Ward-linkage clustering based on the Pearson correlation coefficients) (Fig. 6A). Overall, the AMR profiles of *P. dorei* and *P. vulgatus* were very similar, with 17 of the 23 AMR families present in both species without significant statistical differences. Six AMR families were only found in *P. vulgatus* assemblies. The only AMR family present in higher abundance per genome in *P. dorei* was tetracycline antibiotic resistance, while fluoroquinolone antibiotic/tetracycline antibiotic resistance and aminoglycoside antibiotic resistance were more abundant per genome in *P. vulgatus* (Wilcoxon test, adj. *p* values < 0.05) (Fig. 6C–E). *P. vulgatus* thus had a more diverse yet similar AMR profile than *P. dorei.* Additionally, there were differences in AMR content for strains isolated or present in disease for *P. dorei* and *P. vulgatus* (Supplementary Fig. 2). Of note, fluoroquinolone/tetracycline and glycopeptide antibiotic resistance gene families were higher in strains isolated or present in disease for both species. For *P. vulgatus*, macrolide antibiotic resistance was more abundant in strains isolated or present in disease, while tetracycline antibiotic resistance alone was more abundant in non-disease.

## Discussion

*P. vulgatus* and *P. dorei* are common, abundant and important commensals of the human gut^1,19^. Both species are among the depleted bacteria in CS-born infants and the specific roles and differences between these two species in the human gut and their contribution to health and disease have not yet been explored. In the current study, we investigated, for the first time, the genomic details of 3951 assemblies of *P. vulgatus* and *P. dorei* and performed genomic comparison and phylogenetic analysis to gain insight into the ecology and evolution of these bacteria with high genome plasticity (a summary of key differences can be found in Supplementary Table 2).

We showed that *P. vulgatus* has a bigger pan-genome but smaller genomes overall when compared to *P. dorei*. In other words, *P. vulgatus*, as a species, had a larger collection of genes, but individually, *P. dorei* isolates had a larger collection of genes. Both species had an open pan-genome, indicating a high degree of genome plasticity and adaptability in the context of the human gut. To illustrate this diversity/adaptability, the core genes only represent 1.71% and 0.44% of the pan-genome of *P. dorei* and *P. vulgatus*, respectively, with only a small portion of their genome shared between all the assemblies. Because *P. vulgatus* pan-genome is larger, we thus assume that *P. vulgatus* strains have higher genetic plasticity, in line with^49^. This is confirmed by the higher proportion of ISs, bacteriophages and plasmids in *P. vulgatus*, with concurrently less CRISPR-Cas systems within the genomes, all of which play a role in the bacterial genome instability and driving genome diversification^50^^51^. *P. vulgatus* pan-genome could for example be bigger thanks to the higher number of bacteriophages potentially carrying cargo. Nonetheless, *P. dorei* appears to have had more HGTs, which constitutes a paradox. Looking at the phylogeny and synteny, we hypothesise that *P. dorei* experienced genome expansion directly from a clade of *P. vulgatus*, probably driven by HGT from *Bacteroides* species. Even though *P. dorei* has a higher degree of genome conservation/stability compared to *P. vulgatus*, it could have experienced HGT thanks to ICEs carrying cargo. Since cells need to be in close proximity for ICEs to be transferred, the variety of genes that can be transferred using this mechanism is limited to the genes available in the surrounding environment. Also, although *P. dorei* had more CRISPR-Cas systems, there are other anti-phage defense systems that have not been investigated here and could be prevalent in *P. vulgatus*^52^.

There is further evidence that *P. dorei* evolved directly from a clade of *P. vulgatus*. *P. dorei* is more recent than *P. vulgatus*, as indicated by the fact that *P. vulgatus* was discovered 73 years before *P. dore*i, and it might have undergone recent population bottleneck. *P. dorei* had less genes per unit of genome (Fig. 1F) as well as lower GC content (Fig. 1C), and both could be related to genetic drift^53^^,^^54^. In this case, accumulations of pseudo-genes and mobile genetic elements could be the reasons for less genes per unit of genome. Other explanations could be gene-duplication events, more non-coding regions and genome re-arrangement.

AMR and carbohydrate utilisation are major forces driving bacterial evolution. We showed that there was no significant difference in the AMR profile of *P. dorei* and *P. vulgatus* for most AMR families. Also, *P. dorei* and *P. vulgatus* assemblies did not group separately according to AMR families present in their genome (Fig. 6A). On the other hand, both species grouped separately according to their GH family’s profile, with only a few exceptions (Fig. 5A). These data showed the importance of carbohydrate utilisation and CAZyme profile on species differentiation and evolution. The phylogenetic tree (Fig. 2) showed that a large proportion of the genome assemblies publicly available came from infants’ gut, demonstrating the importance of these two species in early life. We analysed the GH families associated with HMO utilization present in each genome (Fig. 5B). Most *P. dorei* species grouped together, with a few exceptions. *P. dorei* had a higher number of genes associated with HMO utilization present on individual genomes, possibly indicating a better fitness for the infant gut environment than *P. vulgatus*.

### Supplementary Information

Supplementary Figures.

Supplementary Table 1.

Supplementary Table 2.

## Data Availability

All the data used in this manuscript are freely available online. We used the Unified Human Gastrointestinal Genome (UHGG) catalog, deposited in the European Nucleotide Archive under study accession ERP116715 and available from the MGnify FTP site (http://ftp.ebi.ac.uk/pub/databases/metagenomics/mgnify_genomes/). We also used the Early-Life Gut Genomes (ELGG) catalog, deposited in the Zenodo repository under https://doi.org/10.5281/zenodo.6969520. Note that there is no accession number for this catalog, as it was built from previously deposited data with accession numbers listed here: https://static-content.springer.com/esm/art%3A10.1038%2Fs41467-022-32805-z/MediaObjects/41467_2022_32805_MOESM12_ESM.xlsx. Finally, we downloaded genomes of isolates publicly available on the National Center for Biotechnology Information (NCBI) (November 2022): https://www.ncbi.nlm.nih.gov/. All corresponding genomes accession numbers and links are available in Supplementary Table 1.
