# Supplementary material for: Genome plasticity shapes the ecology and evolution of Phocaeicola dorei and Phocaeicola vulgatus
Source: Sci Rep. 2024 May 2;14:10109. doi: 10.1038/s41598-024-59148-7 (PMC11066082; doi:10.1038/s41598-024-59148-7)
Supplement: Supplementary file 1 — Supplementary Figures. [file 41598_2024_59148_MOESM1_ESM.docx]

**Supplementary Figures legends**


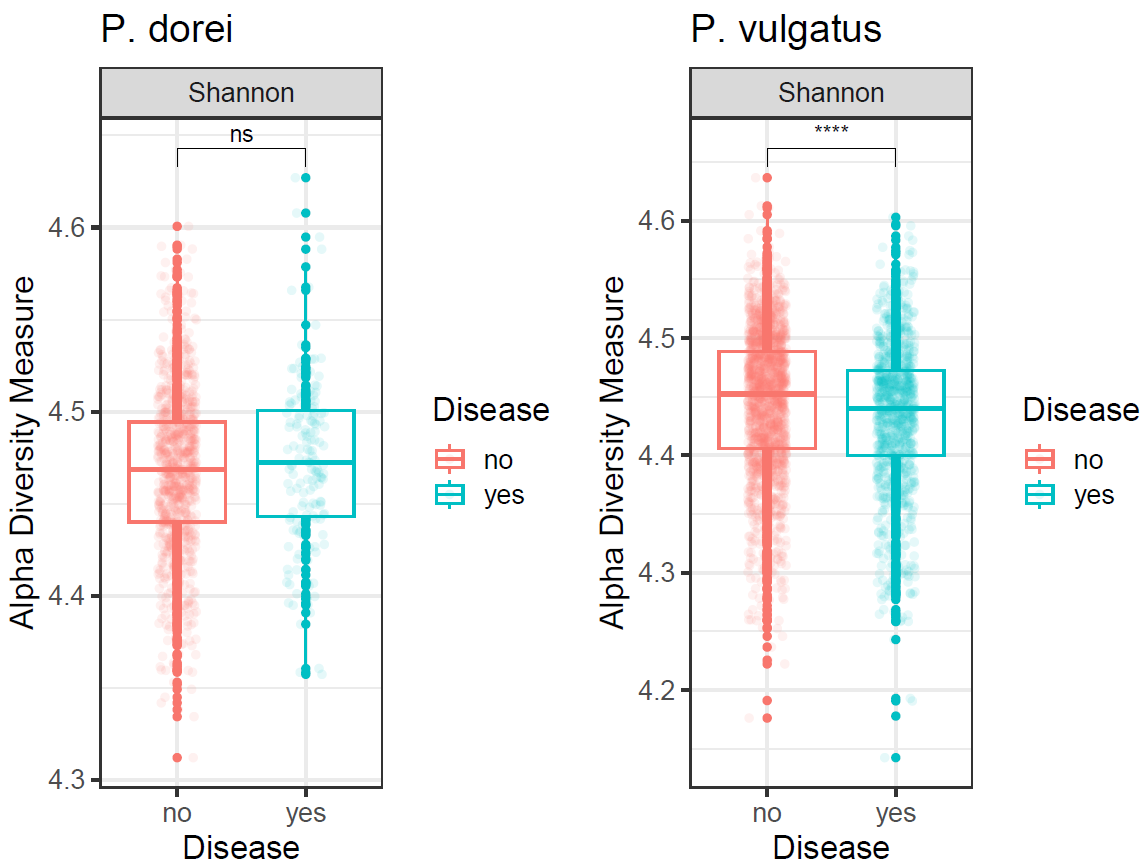


Supplementary Figure 1. Alpha-diversity (Shannon index) comparison for *P. dorei* and *P. vulgatus* strains present or isolated in disease states (yes) or not isolated/present in disease states (no) (Wilcoxon test, ****, adj. p-value<0.0001, ns, non-significant).


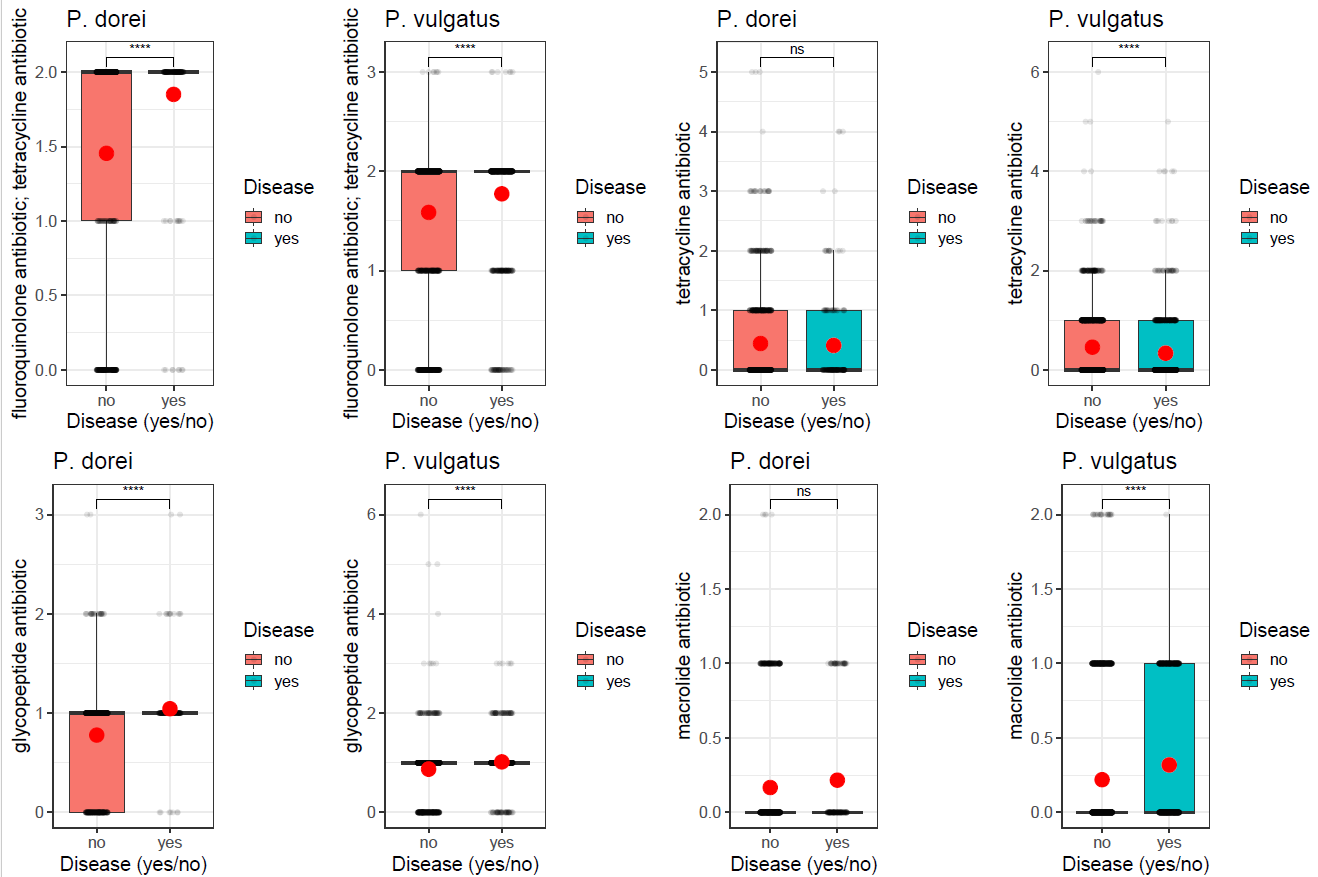


Supplementary Figure 2. AMR comparison (number of genes belonging to a given AMR family per assembly) for *P. dorei* and *P. vulgatus* strains present or isolated in disease states (yes) or not isolated/present in disease states (no) (Wilcoxon test, ****, adj. p-value<0.0001, ***, adj. p-value < 0.001, **, adj. p-value < 0.01, ns, non-significant). Red dots correspond to the average value.
